# A molecular signature for IL-10–producing Th1 cells in protozoan parasitic diseases

**DOI:** 10.1172/jci.insight.169362

**Published:** 2023-12-22

**Authors:** Chelsea L. Edwards, Jessica A. Engel, Fabian de Labastida Rivera, Susanna S. Ng, Dillon Corvino, Marcela Montes de Oca, Teija C.M. Frame, Shashi Bhushan Chauhan, Siddharth Sankar Singh, Awnish Kumar, Yulin Wang, Jinrui Na, Pam Mukhopadhyay, Jason S. Lee, Susanne Nylen, Shyam Sundar, Rajiv Kumar, Christian R. Engwerda

**Affiliations:** 1 QIMR Berghofer Medical Research Institute, Brisbane, Australia.; 2 University of Queensland, School of Medicine, Brisbane, Australia.; 3 Griffith University, School of Natural Sciences, Nathan, Australia.; 4 Institute of Experimental Oncology, University of Bonn, Bonn, Germany.; 5 Institute of Medical Sciences and; 6 Centre of Experimental Medicine and Surgery, Institute of Medical Sciences, Banaras Hindu University, Varanasi, India.; 7 Department of Microbiology, Tumor and Cell Biology, Karolinska Institute, Stockholm, Sweden.

**Keywords:** Microbiology, T cells

## Abstract

Control of visceral leishmaniasis (VL) depends on proinflammatory Th1 cells that activate infected tissue macrophages to kill resident intracellular parasites. However, proinflammatory cytokines produced by Th1 cells can damage tissues and require tight regulation. Th1 cell IL-10 production is an important cell–autologous mechanism to prevent such damage. However, IL-10–producing Th1 (type 1 regulatory; Tr1) cells can also delay control of parasites and the generation of immunity following drug treatment or vaccination. To identify molecules to target in order to alter the balance between Th1 and Tr1 cells for improved antiparasitic immunity, we compared the molecular and phenotypic profiles of Th1 and Tr1 cells in experimental VL caused by *Leishmania donovani* infection of C57BL/6J mice. We also identified a shared Tr1 cell protozoan signature by comparing the transcriptional profiles of Tr1 cells from mice with experimental VL and malaria. We identified LAG3 as an important coinhibitory receptor in patients with VL and experimental VL, and we reveal tissue-specific heterogeneity of coinhibitory receptor expression by Tr1 cells. We also discovered a role for the transcription factor Pbx1 in suppressing CD4^+^ T cell cytokine production. This work provides insights into the development and function of CD4^+^ T cells during protozoan parasitic infections and identifies key immunoregulatory molecules.

## Graphical abstract

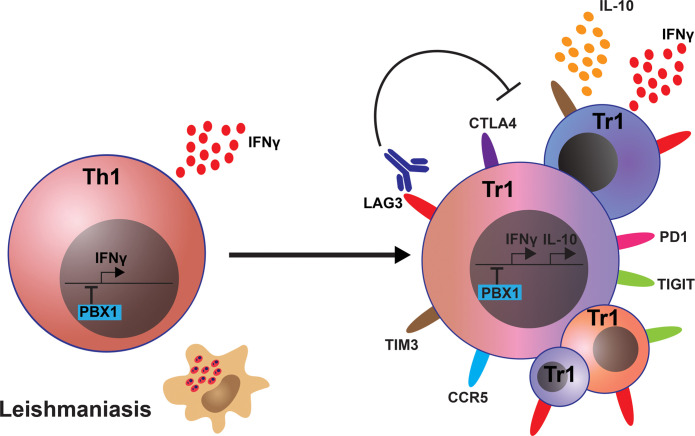

## Introduction

The human immune system primarily evolved to control acute infections in young people, to adjust in order to cope with pregnancy, and to transfer neonatal protection (1, 2). Genes that control immune functions are among the most divergent in modern humans relative to our hominin ancestors, and the major drivers of these changes have been parasites, bacteria, viruses and their products, and the need to control these pathogens and repair damaged tissues (3). Counterbalancing immune regulatory mechanisms developed in parallel to control inflammatory responses and ensure humans survive and reproduce (4). Thus, the maintenance of an intricate balance between pro- and antiinflammatory responses is required to prevent disease. However, established regulatory networks that form a critical counterpoint of this balance can also impede drug- and vaccine-mediated protection against important diseases, such as leishmaniasis, tuberculosis (TB), and malaria (5). This is especially problematic in disease endemic areas.

Following infection, DCs capture and present pathogen antigens to naive CD4^+^ T cells in secondary lymphoid tissues (6). Pattern recognition receptors on DCs trigger cytokine production that stimulates CD4^+^ T cells to develop functional patterns biased toward responses needed to contain and eliminate a particular type of challenge. For example, DC IL-12 production promotes development of proinflammatory T-bet^+^IFN-γ^+^CD4^+^ Th1 cells needed to activate phagocytes to kill captured or resident pathogens in important human diseases, such as those mentioned above: leishmaniasis, TB, and malaria (7–9). There is considerable heterogeneity in the molecules that Th1 cells express, resulting from nuanced pathogen- and tissue-specific adaptions for infection control and prevention of disease (10). However, the inflammatory products produced by Th1 cells to control infection can damage tissues, as may occur in severe manifestations of leishmaniasis, TB, and malaria, as well as in immune-mediated inflammatory diseases such as inflammatory bowel disease (IBD), multiple sclerosis (MS), and type 1 diabetes (11, 12). Th1 cell functions are tightly regulated, and an important way this occurs is by the production of autologous IL-10 that inhibits T cell functions and the activities of antigen presenting cells (APCs) (13). These IL-10–producing Th1 (type 1 regulatory [Tr1]) cells differ from Foxp3-expressing Tregs that are important for maintaining tissue homeostasis (13, 14). They also comprise a substantial proportion of antigen-specific CD4^+^ T cells in patients with visceral leishmaniasis (VL) (15), pulmonary TB (16), and malaria (17). However, in addition to protecting tissues, Tr1 cells may also allow persistent infection by suppressing immune responses via production of antiinflammatory cytokines or expression of coinhibitory receptors (5). Furthermore, they may also restrict responses to vaccination and the effective generation of antiparasitic immunity (5, 7). Therefore, the intricate balance between Th1 and Tr1 cells during infection has a major effect on disease outcome, and the identification of molecules that these CD4^+^ T cell subsets differentially express may provide opportunities to manipulate this balance and promote favorable outcomes in a broad range of human diseases.

Here, we identified gene signatures that distinguished Th1 cells from Tr1 cells in an experimental model of VL caused by infection of C57BL/6J mice with the human parasite *Leishmania donovani*. We also defined a Tr1 cell phenotypic signature for experimental VL and identified candidate molecules to target in order to improve antiparasitic immune responses.

## Results

### Identifying molecules that distinguish Th1 and Tr1 cells in experimental VL.

**Figure 1:**
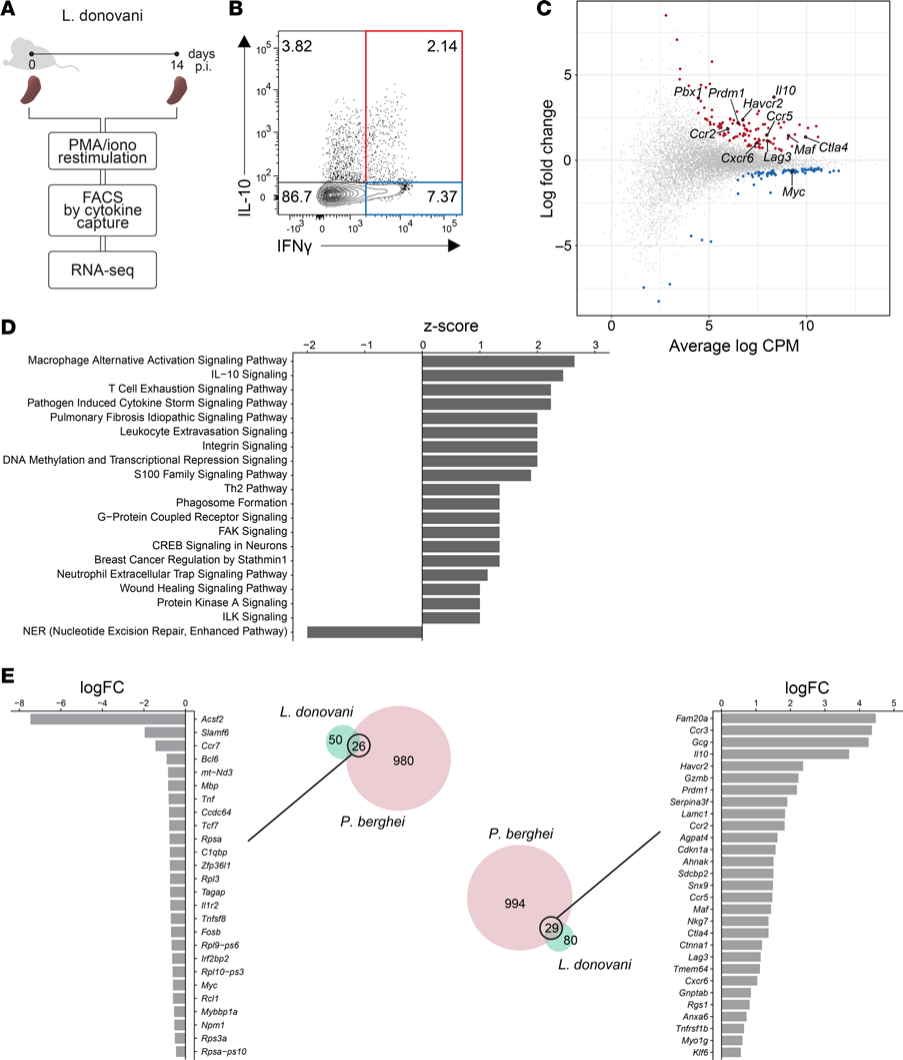
A molecular signature for mouse Tr1 cells during experimental visceral leishmaniasis (VL) caused by *Leishmania donovani*. (**A** and **B**) A schematic showing a brief outline of the work flow for isolating splenic Th1 (IFN-γ^+^ IL-10^–^; blue quadrant) and Tr1 cells (IFN-γ^+^ IL-10^+^; red quadrant). (**C**) Mean-difference plot from differential gene expression analysis between Tr1 versus Th1 cells. The 109 upregulated differentially expressed genes (DEGs) are colored red, the 76 downregulated DEGs are coloured blue, and nonsignificant genes are coloured gray. (**D**) The up- and downregulated canonical pathways identified in Tr1 cells, relative to Th1 cells, from Ingenuity Pathway Analysis are listed. (**E**) DEGs from experimental VL Tr1 versus Th1 cell comparisons were compared with 2,031 DEGs in experimental *Plasmodium berghei* ANKA Tr1 cells versus Th1 cells. The Venn diagrams show that 26 and 29 Tr1 cell DEGs were commonly down- and upregulated, respectively, in these 2 infections. Log fold change values shown are from experimental VL Tr1 cells versus Th1 cells.

To identify how Th1 and Tr1 cells can be distinguished at the molecular level and to understand how such differences may contribute to their unique functional roles, we first used cytokine capture to isolate cells. This was based on IFN-γ and IL-10 expression for Th1 (CD4^+^IFN-γ^+^IL-10^–^) and Tr1 (CD4^+^IFN-γ^+^IL-10^+^) cells from the spleens of C57BL/6J mice infected with *L*. *donovani* at day 14 postinfection (p.i.) (Figure 1, A and B, and Supplemental Figure 1; supplemental material available online with this article; https://doi.org/10.1172/jci.insight.169362DS1). We chose this time point because we previously showed that it coincided with peak Tr1 cell responses in this model (18, 19). We identified differentially expressed genes (DEGs) between Th1 and Tr1 cells using RNA-Seq (Supplemental Table 1), and these included a number of coinhibitory receptors, chemokine receptors, and transcription factor DEGs (Figure 1C). In total, 185 DEGs between Tr1 and Th1 cells were identified, with 109 and 76 significantly up- and downregulated, respectively. Upregulated DEGs in Tr1 cells, relative to Th1 cells, included those encoding the coinhibitory receptor molecules *Ctla4*, *Havcr2* (encoding TIM3), and *Lag3*; chemokine receptors *Ccr2*, *Ccr5*, and *Cxcr6*; and transcription factors *Prdm1*, *Maf*, and *Pbx1* (Figure 1C and Supplemental Table 1). Ingenuity pathway analysis of the data set led to identification of major upregulated canonical pathways, including those associated with IL-10 signaling and T cell exhaustion, while the only downregulated canonical pathway was associated with nucleotide excision repair (Figure 1D).

To identify a molecular signature shared between different protozoan parasite infections, we compared our experimental VL Tr1 cell molecular signature with one we previously reported from experimental malaria infection (20) (Figure 1E and Supplemental Table 2). We performed comparisons between DEGs identified between Tr1 and Th1 cells in experimental VL to 2,031 DEGs in Tr1 versus Th1 cells in experimental malaria (*Plasmodium berghei* ANKA). We excluded genes that were differentially expressed in opposing directions in the 2 data sets based on log fold change (logFC). From this, we identified 26 common DEGs downregulated in Tr1 cells from both parasitic diseases, including *Ccr7*, *Tcf7*, and *Bcl6*, as well as 29 upregulated DEGs, including *Il10*, *Havcr2*, *Prdm1*, *Ccr2*, *Ccr5*, *Maf*, and *Lag3* (Figure 1E).

### Expression of coinhibitory receptor molecules on Th1 and Tr1 cells.

**Figure 2:**
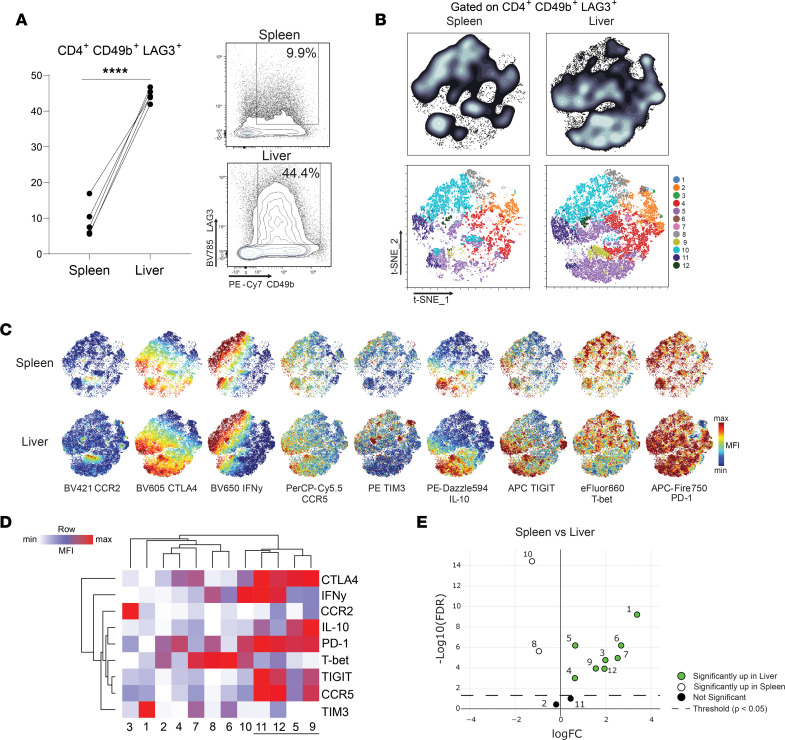
Development of coinhibitory receptor–rich CD4^+^ T cells in the liver during experimental visceral leishmaniasis caused by *Leishmania donovani*. (**A**) Spleen and liver Tr1 cells defined by high levels of CD49b and LAG3 expression at day 14 after infection. (**B**) tSNE and flow self-organizing maps were used for clustering of Tr1 cells identified in **A**, based on the mean fluorescence intensity (MFI) of CCR2, CTLA-4, IFN-γ, CCR5, TIM3, IL-10, TIGIT, T-bet, and PD-1. (**C**) tSNE plots showing the expression of each marker. (**D**) A heatmap showing the expression (MFI) of individual markers in each cluster, with the coinhibitory receptor–rich clusters among Tr1 cells underlined. (**E**) EdgeR was used to establish significant differences in the frequencies of each Tr1 cell cluster in the spleen and liver. *n* = 5 paired tissue samples; *P* < 0.05. ****P < 0001 as determined by a 1-tailed paired *t* test. Significance was assessed by edgeR.

We next sought to confirm protein level expression of selected Tr1 cell–associated coinhibitory receptors and chemokine receptors in mice infected with *L*. *donovani*. Given the increased expression of *Lag3* by Tr1 cells reported above and previous reports classifying Tr1 cells as CD4^+^FOXP3^–^LAG3^+^CD49b^+^ (21, 22), this phenotype was used to subsequently define these cells in lieu of IFN-γ and IL-10 expression (Figure 2A and Supplemental Figure 2A). In support of this characterization, we confirmed that the majority of splenic CD4^+^IFN-γ^+^IL-10^+^ cells exhibited this CD4^+^FOXP3^–^LAG3^+^CD49b^+^ profile in *L*. *donovani*–infected B6.*Il10gfp* × *Ifngyfp* × *foxp3rfp* mice at day 14 p.i. (Supplemental Figure 3). However, it is important to note that these Tr1 cells were highly heterogeneous in their expression of coinhibitory receptors (Figure 2, B and C). Nevertheless, this strategy allowed for the identification of Tr1 cells without the need for PMA and ionomycin restimulation required for detection of IL-10, which can potentially result in altered expression of certain cell-surface molecules. In addition to markers represented within the above DEGs, PD-1 and TIGIT were also examined, due to their association with Tr1 cell function in intestinal inflammation (21) and the established role for PD-1 in immune regulation in VL (23, 24). Furthermore, analysis was extended to include the liver, in which an effective CD4^+^ T cell–dependent response develops to control parasitic growth, in contrast to the spleen where chronic infection is established (25).

In *L*. *donovani*–infected mice, the frequency of Tr1 cells among CD4^+^ T cells in the liver was around 4-fold higher than in the spleen at day 14 p.i. (Figure 2A). Furthermore, the expression of CCR2, CTLA-4, IFN-γ, CCR5, TIM3, IL-10, TIGIT, T-bet, and PD-1 was higher on splenic and hepatic Tr1 cells, compared with all other CD4^+^ T cells (Supplemental Figure 2B). Given the molecular and functional heterogeneity previously reported among Tr1 cells (21), we next examined the heterogeneity of expression of the above coinhibitory receptors and chemokine receptors on these cells from the spleen and liver after infection. Following t-distributed Stochastic Neighbor Embedding (tSNE) clustering of Tr1 cells, we could also identify Tr1 cells expressing high levels of the majority of coinhibitory receptors (coinhibitory receptor rich), as well as those expressing fewer coinhibitory receptors (coinhibitory receptor poor) in the spleen and liver (Figure 2, B, C, and D). However, a greater frequency of coinhibitory receptor–rich CD4^+^ T cell clusters were found in the liver, where infection was better controlled, compared with the spleen, where a chronic infection would be established (Figure 2E). Thus, *L*. *donovani* infection in mice was associated with the development of Tr1 cells in both the spleen and liver, but the liver contained increased frequencies of this CD4^+^ T cell subset and more population complexity in terms of patterns of coinhibitory receptor expression.

### LAG3 blockade suppressed Tr1 cell development and improved antiparasitic immunity.

**Figure 3:**
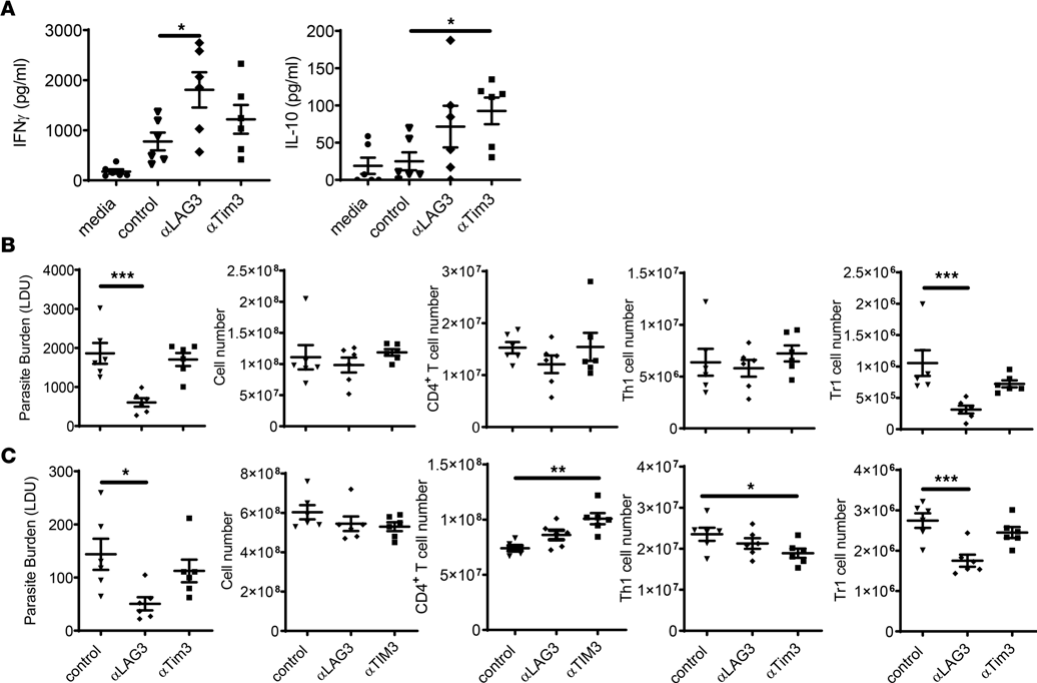
LAG3 blockade improves antiparasitic immunity during *Leishmania donovani* infection. (**A**) Spleen cells from mice infected for 28 days with *L*. *donovani* were stimulated with parasite antigen for 72 hours in the presence of anti-LAG3 (diamonds), anti-TIM3 (squares), or control (triangles) mAbs, and IFN-γ and IL-10 production were measured, as indicated. (**B** and **C**) Mice infected with *L*. *donovani* for 14 days were treated with anti-LAG3, anti-TIM3, or control mAb for a further 14 days prior to measuring liver (**B**) and spleen (**C**) parasitic burdens. Associated cellular responses at 28 days after infection are shown. *n* = 6 mice per group. Experiments were conducted twice. Data are shown as mean ± SEM; *P < 0.05, **P < 0.01, and ***P < 0.001. Significance was assessed by 1-way ANOVA.

The blockade of several coinhibitory receptor molecules has shown therapeutic value in experimental VL, including CTLA-4 and PD-1 (23, 24, 26, 27). However, the therapeutic potential of LAG3 and TIM3 blockade in VL is unknown. We chose to focus on these coinhibitory receptors because (a) they were both differentially expressed between Tr1 and Th1 cells (Figure 1C), (b) LAG3 has a strong association with Tr1 cells and their functions (14), and (c) TIM3 loss-of-function mutations are associated with human diseases and it has emerged as an important target for immune checkpoint blockade in trials to treat several different human cancers (28). We first measured the effects of mAb-mediated LAG3 or TIM3 blockade on cytokine production in an ex vivo antigen restimulation assay using spleen cells from mice at 28 days p.i. with *L*. *donovani*. We chose this time point because we know LAG3 and TIM3 were expressed by day 14 p.i. (Figure 2), and a chronic infection then becomes established in the spleen by day 28 p.i. (29). LAG3, but not TIM3, blockade resulted in a 2-fold increase in average IFN-γ production in cell culture supernatants, compared with control samples (Figure 3A). Although IL-10 levels were also elevated following LAG3 or TIM3 blockade, this increase only reached statistical significance following TIM3 blockade (Figure 3A). We next examined the effect of LAG3 or TIM3 neutralization on antiparasitic immunity in vivo. Mice infected with *L*. *donovani* for 14 days were administered anti-LAG3, anti-TIM3, or control mAb via i.p. injection every 3 days for a further 14 days. Mice that receive LAG3, but not TIM3, blockade exhibited reduced liver (Figure 3B) and spleen (Figure 3C) parasitic burdens, associated with a decrease in Tr1 cell numbers, relative to control mice. We observed a small increase in splenic CD4^+^ T cells numbers following LAG3 or TIM3 blockade, but this only reached statistical significance in mice receiving anti-TIM3 mAb (Figure 3C). We also found no significant changes in Th1 cell numbers in the liver or spleen following LAG3 blockade, while there was a small decrease in the spleen after TIM3 blockade (Figure 3C). It should be noted that the anti-LAG3 mAb (clone C9B7W) is nondepleting and acts by blocking the interactions between LAG3 and its functional ligands (30–32). Thus, LAG3 blockade improved antiparasitic immunity in association with reduced numbers of Tr1 cells and a limited effect on the inflammatory response, as indicated by a marginal change in Th1 cell numbers at sites of infection. In contrast, TIM3 neutralization did not appear to alter antiparasitic immunity.

**Figure 4:**
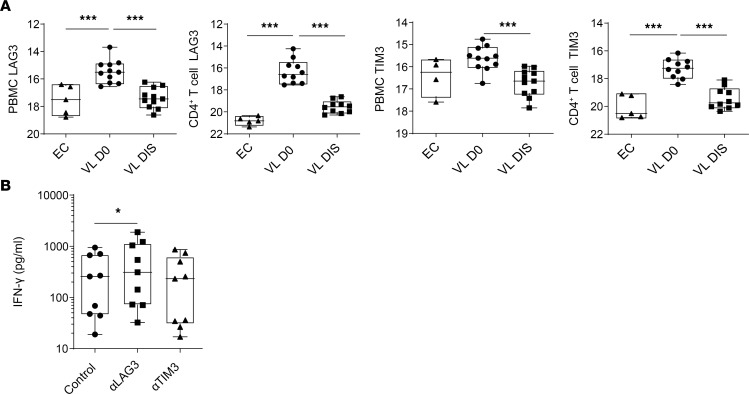
LAG3 blockade improves antiparasitic immunity during visceral leishmaniasis. (**A**) LAG3 and TIM3 expression was measured by qPCR in PBMCs and CD4^+^ T cells from blood samples from patients with visceral leishmaniasis (VL) on admission to clinic for antiparasitic drug treatment (VL D0), from the same patients 30 days later at discharge from clinic (VL DIS) (*n* = 10), and from endemic controls (EC) (*n* = 5), as indicated. Box and whisker plots show the median of the data points as well as the minimum and maximum of the data points; ***P < 0.001. Significance was assessed by Mann-Whitney *U* test. (**B**) Antigen-specific IFN-γ production was measured in whole-blood cells from patients with VL (*n* = 9) cultured for 24 hours with soluble *leishmania* antigen (SLA) with anti-LAG3, anti-TIM3, or control mAb, as indicated. Box and whisker plots show the median of the data points as well as the minimum and maximum of the data points; *P < 0.05. Significance was assessed by Wilcoxon matched-pairs signed rank test.

We next sought to determine the potential of LAG3 and TIM3 as clinical targets in human parasitic disease. *LAG3* and *TIM3* mRNA expression was assessed in patients with VL, with increased accumulation of *LAG3* observed in PBMCs and CD4^+^ T cells from patients on admission to clinic for antiparasitic drug treatment (VL day 0 [D0]), compared with samples obtained from the same patients 30 days later, after discharge (VL DIS), and those from endemic controls (EC) (Figure 4A). A similar pattern of expression was also noted for *TIM3* (Figure 4A). A whole-blood assay was then employed to examine the effects of LAG3 or TIM3 blockade on antigen-specific cellular responses in patients with VL. IFN-γ production was increased following LAG3, but not TIM3 blockade, compared with blood cells treated with an isotype control mAb (Figure 4B). Together, these results highlight the therapeutic potential of targeting LAG3, but not TIM3, to improve proinflammatory cytokine production and limit the expansion and immunoregulatory function of Tr1 cells during established *L*. *donovani* infection.

### PBX1 binds the Il10 and Ifng promoters in Tr1 cells.

**Figure 5:**
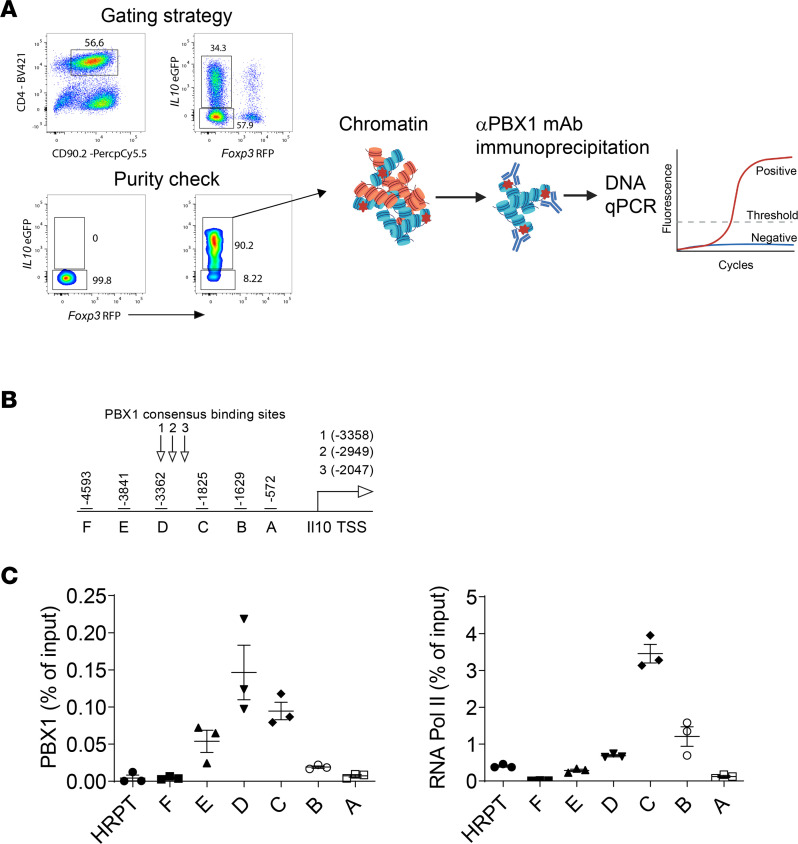
PBX1 binds the *Il10* promoter in Tr1 cells. (**A**) Tr1 cells were sorted from mouse splenocytes cultured in Tr1 cell polarizing conditions. Tr1 cells were assessed via ChIP for PBX1 and RNA Pol II binding upstream of the *Il10* transcription start site (TSS) in the promoter region. (**B**) PBX1 consensus binding sites in the *Il10* promoter are indicated. (**C**) Recruitment of PBX1 and RNA Pol II across the *Il10* promoter (regions A–F) and in a control region of the genome (*Hprt*) are shown.

PBX1 is a transcriptional regulator that modulates the DNA-binding function of homeobox proteins (33). It was among the 185 DEGs identified between Tr1 and Th1 cells (Figure 1C) and has previously been shown to regulate IL-10 production by macrophages following interactions with apoptotic cells (34). To examine whether PBX1 might be involved in *Il10* gene transcription in CD4^+^ T cells, murine Tr1 cells were generated in vitro, and ChIP PCR was performed using an anti-PBX1 mAb and PCR primers designed to amplify DNA in the *Il10* promoter (Figure 5A). Analysis of the *Il10* promoter revealed 3 potential PBX1 consensus binding sites (Figure 5B), and ChIP PCR demonstrated that PBX1 bound at these sites (Figure 5C). Additionally, RNA polymerase II (RNA Pol II) was found to bind in relative close proximity to the PBX1 binding sites (Figure 5C). We also interrogated the *Ifng* promoter and found several potential PBX1 consensus binding sites but identified only 1 region where PBX1 bound; again, this was in relative close proximity to RNA Pol II binding sites (Supplemental Figure 4). Thus, PBX1 interacts with the *Il10* and *Ifng* promoters in Tr1 cells in close proximity to sites that are transcriptionally active.

### Pbx1 restricts proinflammatory cytokine production by T cells during infection.

**Figure 6:**
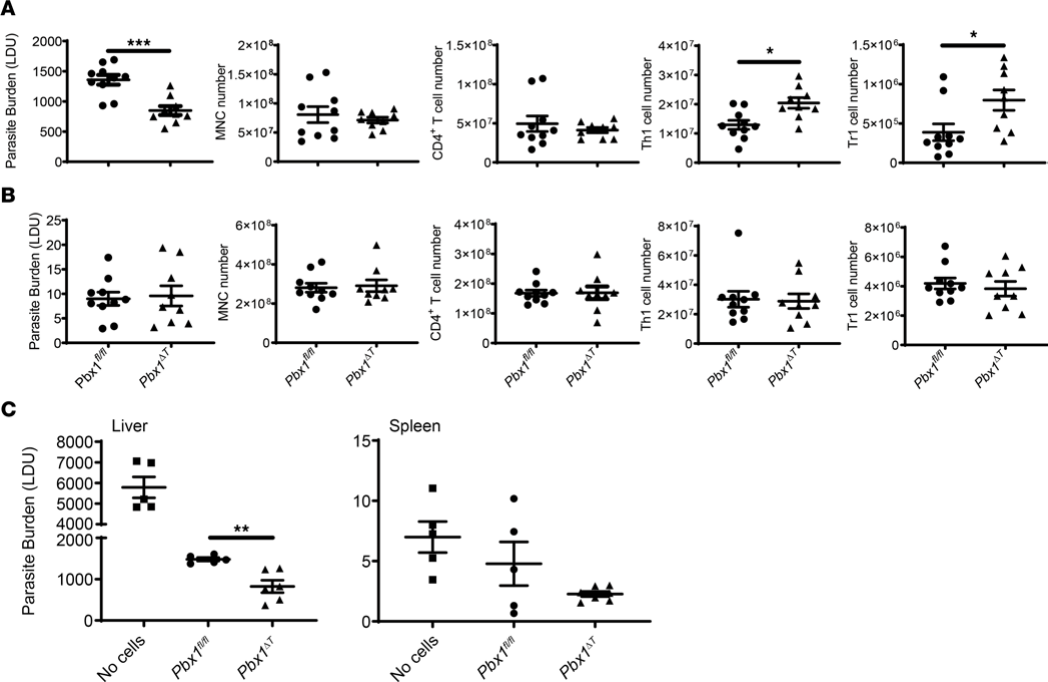
PBX1 inhibits parasite control and CD4^+^ T cell inflammatory responses. (**A** and **B**) *Pbx1*^ΔT^ (*n* = 10) and littermate control mice (*Pbx1^fl/fl^*; *n* = 9) were infected with *Leishmania donovani* for 14 days prior to measuring parasitic burdens and associated cellular responses in the liver (**A**) and spleen (**B**). The gating used to define Th1 and Tr1 cells is shown in Supplemental Figure 2. Data pooled from 2 independent experiments. Data are shown as mean ± SEM; *P < 0.05 and ***P < 0.001. Significance was assessed by Mann-Whitney *U* test. (**C**) Liver and spleen parasitic burdens were measured in B6.*Rag1*^–/–^ mice infected with *L*. *donovani* for 14 days and in mice that received CD4^+^ T cells isolated from either *Pbx1*^ΔT^ or *Pbx1^fl/fl^* mice. *n* = 5–6 mice per group. The experiment was conducted twice. Data are shown as mean ± SEM; **P < 0.01. Significance was assessed by Mann-Whitney *U* test.

To study the role of PBX1 in T cells during infection, T cell–specific *Pbx1*-deficient (*Pbx1*^ΔT^) mice were generated by crossing *Cd4*-*Cre* transgenic mice (35) with *Pbx1^fl/fl^* mice (36). We then examined the effect of T cell–specific *Pbx1* deficiency in experimental VL and found that *Pbx1*^ΔT^ mice exhibited a significant, 2-fold reduction in hepatic parasitic burdens, compared with *Cre*^–^ littermate control (*Pbx1^fl/fl^*) mice, at day 14 p.i. with *L*. *donovani* (Figure 6A). However, rather than displaying diminished Tr1 cell numbers, as might be predicted from the above RNA-Seq data, these animals had significantly higher numbers of both Th1 and Tr1 cells in the liver, compared with littermate controls (Figure 6A). We found no change in steady state levels (no stimulation) of *Ifng* or *Il10* mRNA in *Pbx1*^ΔT^ CD4^+^ T cells, compared with corresponding cells from *Pbx1^fl/fl^* mice (Supplemental Figure 5A). No changes in parasitic burden or T cell responses were found in the spleens of these animals (Figure 6B), indicating a tissue-specific effect of T cell–specific *Pbx1* deficiency at the day 14 p.i. time point examined. This was supported by increased IFN-γ and IL-10 production by hepatic *Pbx1*^ΔT^, relative to *Pbx1^fl/fl^* CD4^+^ T cells (Supplemental Figure 5B), but no difference in these cells in the spleen (Supplemental Figure 5C).

To confirm that the described changes in hepatic antiparasitic immunity were caused by CD4^+^ T cells, *Pbx1*^ΔT^ or *Pbx1^fl/fl^* CD4^+^ T cells were adoptively transferred into B6.*Rag1*^–/–^ mice prior to challenge with *L*. *donovani*. While B6.*Rag1*^–/–^ mice that received CD4^+^ T cells from either source had substantially better control of parasitic growth compared with animals receiving no cells, B6.*Rag1*^–/–^ mice that received *Pbx1*^ΔT^ CD4^+^ T cells had significantly lower (again, approximately 2-fold) hepatic parasitic burdens than mice that received control *Pbx1^fl/fl^* CD4^+^ T cells at day 14 p.i. (Figure 6C). Although a reduction in splenic parasitic burden was apparent in B6.*Rag1*^–/–^ mice that received *Pbx1*^ΔT^ CD4^+^ T cells, this change was not significantly different than a change from mice that received control *Pbx1^fl/fl^* CD4^+^ T cells. Thus, these data demonstrate that *Pbx1*-deficient CD4^+^ T cells possess enhanced antiparasitic activity. Furthermore, these results indicate that, although *Pbx1* expression was higher in Tr1 cells relative to Th1 cells in our transcriptional studies (Figure 1C), it played a limited role, if any, in the generation and/or maintenance of Tr1 cells. Rather, we discovered that PBX1 suppressed Th1 cell development and possibly the subsequent transition to Tr1 cells during experimental VL.

## Discussion

In this study, we identified a molecular signature for Tr1 cells that distinguished them from Th1 cells in experimental VL, a disease model for a parasitic disease that causes substantial morbidity and mortality among vulnerable people living in disease endemic areas. We showed that Tr1 cells are a heterogeneous CD4^+^ T cell subpopulation during experimental VL, based on coinhibitor receptor expression, with a more complex population composition in the liver compared with the spleen. We demonstrated that targeting LAG3 with neutralizing mAb during established, experimental *L*. *donovani* infection promoted antiparasitic immunity in the liver. We also examined the Tr1 cell–associated transcription factor PBX1 and found that it suppressed proinflammatory cytokine production by activated CD4^+^ T cells at sites of infection and inflammation.

Through their antiinflammatory functions (13, 14, 22), Tr1 cells play an important role in mitigating tissue damage from sustained inflammation during infection. However, this regulation can also result in prolonged pathogen persistence and, thereby, disease maintenance or exacerbation (19). Previous studies have shown that Tr1 cells develop early during infection (15, 17, 37–41), have a marked influence on disease outcome, and can hamper vaccine efficacy (5). Although Tr1 cells are known to differentiate from effector CD4^+^ T cell subsets, such as Th1 cells (42), the molecular mechanisms governing their differentiation and their long-term maintenance are not clear. We have previously shown that Tr1 cells are antigen specific in experimental VL by using the surrogate activation markers CD49d and CD11a (19, 30), as well as a MHC II I-A^b^ PEPCK_335-351_ tetramer that recognizes CD4^+^ T cells reactive against a naturally processed immunodominant antigen derived from *Leishmania* glycosomal phosphoenol-pyruvate carboxykinase (PEPCK) conserved in all pathogenic *Leishmania* species (18, 43). Thus, accumulating evidence suggests that antigen-specific Tr1 cells develop either soon after or in parallel with corresponding effector CD4^+^ T cell populations as a mechanism to control the extent and magnitude of these latter CD4^+^ T cell responses during infection.

Recent studies have identified extensive molecular and phenotypic heterogeneity in Tr1 cell populations (21, 44). Our analysis of Tr1 cells from mice infected with *L*. *donovani* aligns with these findings, whereby we identify both coinhibitory receptor–rich and –poor subpopulations. Furthermore, during experimental VL, we recorded considerable tissue-specific disparities in the frequency and distribution of these Tr1 cell subsets. The increased frequency of Tr1 cells in the liver, relative to the spleen, suggests that these cells may play an important role in maintaining an appropriate balance between pro- and antiinflammatory responses that enables effective antiparasitic immunity to progress. The lower frequency of Tr1 cells in the spleen is associated with development of TNF-mediated tissue destruction that impairs CD4^+^ T cell trafficking into this organ (29), and this has been shown to be exacerbated when T cell IL-10 is limited (19). These findings are important for understanding the use of coinhibitory receptor or antiinflammatory cytokine blockade as therapeutics, and they may explain some of the variability seen in individual responses, as well as the improved responses observed when a combination of coinhibitory receptor-blocking measures are employed.

LAG3 was one of the first coinhibitory receptors to be associated with Tr1 cell immunoregulatory function (22). It is structurally homologous to CD4 and, as such, can bind MHC II, thereby potentially suppressing T cell activation via the inhibition of CD4 engagement (32, 45). However, this suppressive activity has also been shown to occur via binding of stable peptide MHC II complexes (46). Furthermore, Fibrinogen-like protein 1 was identified as a major inhibitory ligand for LAG3 (47), thus highlighting an alternative suppressive pathway mediated by this coinhibitory receptor. Although we didn’t identify the key functional ligand for LAG3 in our studies of experimental VL, we were able to show that LAG3 blockade had an effect on disease outcome. This effect, however, was restricted to the liver, despite increased LAG3 expression by splenic Tr1 cells being detected. Recent evidence indicates that LAG3 functions as a signal disrupter in the TCR immune synapse by causing disassociation of the tyrosine kinase Lck from the CD4 and CD8 coreceptors (48). One possible explanation for the greater effect of LAG3 blockade in the liver compared with the spleen is the increased proportion of activated, antigen-specific CD4^+^ T cells in the liver relative to the spleen, including Tr1 cells (49). In this study, we also show that the frequency of coinhibitory receptor–rich Tr1 cells was greater in the liver than the spleen. Thus, greater signaling via the TCR may have made hepatic Tr1 cell development and functions more vulnerable to LAG3 blockade. One limitation of our studies on LAG3 was, despite the anti-LAG3 mAb (clone C9B7W) we employed being reported as non–cell depleting, we did not test whether this mAb was cell depleting in our experimental model and, therefore, cannot rule out the possibility that some LAG3^+^ cells, including Tr1 cells, may have been depleted. Furthermore, because LAG3 can be expressed by cell populations other than Tr1 cells, we cannot rule out the possibility that at least some of the therapeutic effects we observe resulted from modulation of other immune cell populations. Recently, we found that PD-1 blockade enhanced antigen-specific IFN-γ and IL-10 production in PBMCs isolated from volunteers participating in controlled human malaria infection (CHMI) studies following activation with *P*. *falciparum* antigen (50). Of note, CTLA-4, LAG3, or TIM3 blockade in the same study failed to alter parasite-specific responses. Previous work in a mouse model of malaria caused by *Plasmodium falciparum* *yoelii* 17XNL found combined neutralization of PD-1 and LAG3 signaling was required to enhance antiparasitic immunity (30). Together with our finding that LAG3 blockade alone improved antiparasitic immune responses in experimental VL and samples from patients with VL, whereas TIM3 blockade did not, these data support the notion that the most effective coinhibitory receptor targets are likely to vary between different diseases. Thus, while LAG3 blockade improved antiparasitic immunity in VL, this strategy required combination with PD-1 blockade in the context of *Plasmodium* infection, and further differences are likely to be found for other disease settings.

PBX1 is a homeobox protein that has previously been shown to bind the *Il10* promoter in macrophages and drive gene transcription following association with apoptotic cells (34). However, while PBX1 bound the *Il10* promoter in Tr1 cells around the same location as RNA Pol II, we found no evidence of reduced IL-10 levels in *Pbx1*-deficient Tr1 or CD4^+^ T cells. In fact, we often found increased CD4^+^ T cell IL-10 production, indicating that PBX1 was not required to promote IL-10 production by CD4^+^ T cells during experimental VL. Instead, PBX1 appeared to play a role in limiting cytokine production by CD4^+^ T cells. One possible explanation for our findings in mice with *Pbx1*-deficient T cells is that PBX1 acts as a pioneer transcription factor for multiple cytokine genes, as has previously been reported for *Myogenin* and estrogen receptor α (*ERα*) target genes (51–53). With regard to the latter genes, PBX1 was also identified as a partner pioneering factor for FOXA1, and although their functions were independent, they both had a synergistic effect on chromatin openness where they bound (52). Although we identified *Pbx1* as a differentially expressed Tr1 cell gene in experimental VL, it was among the lowest transcribed genes identified in our study. Therefore, our identification of PBX1 as a potent immune regulator in CD4^+^ T cells may have been serendipitous, although further work on PBX1 protein levels and cellular localization is required before further conclusions can be made on the cell-specific role of this transcriptional regulator.

We have previously shown that Tr1 cells emerge rapidly during infection and play important roles in limiting tissue pathology due to inflammation, yet they also inhibit antiparasitic immunity during VL (15, 19, 54). Therefore, any manipulation of Tr1 cell development or functions must consider the potential negative consequences. In the studies presented here, we observed no gross changes in hepatosplenomegaly, overall immune cell numbers, or evidence of increased tissue necrosis following blockade of LAG3 or TIM3. We also observed none of these changes following deletion of *Pbx1* in the CD4^+^ T cell compartment, indicating that these immune modulatory strategies were not causing tissue damage. We previously reported that disruption of IL-10 production by T cells during experimental VL caused TNF-mediated destruction of splenic tissue structure (18, 19, 55). However, in the case of LAG3 blockade, despite reduced Tr1 cell numbers, we didn’t find a corresponding increase in proinflammatory signal. In regard to T cell–specific deletion of *Pbx1*, our findings indicate a shared effect on both pro- and antiinflammatory responses. Thus, under these immunomodulatory conditions, the balance between antiparasitic immune and immune regulatory responses appeared to be maintained. Nevertheless, the increased IFN-γ production following stimulation of VL patient blood cells with parasite antigen indicates that caution must be applied when planning such host-directed interventions in humans.

VL in India is caused by *L*. *donovani*, which has evolved with humans for millennia (56). Consequently, the majority of infections (~90% in India), remain asymptomatic (57), with a balanced antiparasitic immune response that controls parasites and prevents disease. However, immune responses in infected individuals that progress to VL are unable to do this (5, 7, 25, 58), and the balance of Th1 and Tr1 cell responses plays a critical role in determining this outcome (5, 7). Given the requirement of the host immune system for optimal responses to the antiparasitic drugs used to treat VL, as well as other parasitic diseases (59, 60), manipulating Tr1 cells in the context of drug treatment represents a potential strategy to improve antiparasitic immunity in people living in areas endemic for parasitic diseases. In the case of VL, where sterile cure is unlikely (61–63), this approach may also help reduce the risk of parasite transmission by keeping persisting parasite numbers low enough so parasites are unlikely to be taken up by insect vectors; it may help to reduce the risk of developing disease complications such as post kala-azar dermal leishmaniasis. The Tr1 cell–associated molecules identified in our study are potential targets for immune modulation, applicable to any disease in which Tr1 cells play a pivotal role.

Overall, we show that Tr1 cells are composed of a heterogeneous cell population in experimental VL, characterized by the expression of coinhibitory receptors, chemokine receptors, and transcription factors. We identified a Tr1 cell–associated gene signature distinguishing this subset from Th1 cells. These findings have important implications for the identification of molecular targets to manipulate Tr1 cell responses for improved disease outcomes.

## Methods

### VL patient samples.

All patients with VL were diagnosed by detection of antibodies in serum reactive against recombinant K39 and/or amastigotes in the splenic biopsies at Kala-Azar Medical Research Centre (KAMRC), Muzaffarpur, Bihar, India. Aggregate clinical data are presented in Supplemental Table 3. Heparinized venous blood was collected from infected patients (*n* = 30) and endemic healthy controls (*n* = 12). All patients were HIV^–^ and older than 12 years.

### Processing human blood.

Peripheral blood mononuclear cells (PBMCs) were isolated from human blood samples by Ficoll-Paque (GE Healthcare Life Sciences) gradient centrifugation at 300*g* at room temperature for 25 minutes. In some cases, CD4^+^ cells were isolated from PBMCs by MACS purification using CD4 (clone L3T4) MicroBeads (Miltenyi Biotec) according to the manufacturer’s guidelines. PBMCs were stored at –80°C in 10% DMSO/90% FCS prior to analysis. In some cases, whole-blood assays were performed on fresh VL patient blood to measure antigen-specific IFN-γ production, as previously described (64). Anti-LAG3 mAb (17B4; Enzo Life Science), anti-TIM3 (QA20A20; BioLegend), or isotype control mAb (MOPC-21; mouse IgG1κ; BioLegend) were added to cultures at 10 μg/mL.

### Mice.

Female mice between 6 and 12 weeks of age were used for all experiments. Mice were group housed with a maximum of 6 mice per cage and maintained under pathogen-free conditions at the QIMR Berghofer Medical Research Institute Animal Facility. C57BL/6J (RRID: IMSR_JAX:000664) mice were sourced from the Walter and Eliza Hall Medical Research Institute (Kew VIC, Australia). All other mice were bred in-house, including C57BL/6*-Foxp3^tm1flv^*/J (Foxp3-RFP; JAX:008374) (65), C57BL/6-*Il10^tm1Flv^*/J (IL-10–GFP; JAX:008379) (66), B6.129S4-*Ifng^tm3.1Lky^*/J (*Ifng*YFP, RRID: IMSR_JAX: 017581) (67), B6.*Pbx1^tm3.1Mlc^*/J (*Pbx1^fl/fl^*; JAX:013085) (36), B6.Cg-Tg1^Cwi^/J (*Cd4*-*Cre*; JAX:022071) (35), and B6.129S7-*Rag1^tm1Mom^*/J (*Rag1*^–/–^; JAX:002216) (68). T cell–specific *Pbx1*-deficient mice were generated by crossing B6.*Cd4*-*Cre* transgenic mice with *Pbx1^fl/fl^* mice, thus generating *Pbx1*^ΔT^ animals. In all experiments with these mice, *Cre*^–^ (*Pbx1^fl/fl^*) littermates were used as controls.

### L. donovani infections in mice.

Female C57BL/6J mice, 6–10 weeks old, were purchased from the Australian Resource Centre (Canning Vale, Western Australia, Australia) and the Walter and Eliza Hall Institute (Melbourne, Victoria, Australia). *L*. *donovani* (LV9; MHOM/ET/67/HU3) (69) was maintained by passage in B6*.Rag1^–/–^* mice. Parasites were isolated from the spleens of infected passage mice, and 2 × 10^7^ amastigotes were injected i.v., as previously described (70). Parasitic burden was measured from tissue impressions and expressed in Leishman-Donovan units (LDU) as previously described (19). Spleens were taken for processing and cellular analysis. In some experiments, CD4^+^ T cells were isolated from mouse spleens, and 1 × 10^6^ of these cells were transferred into B6.*Rag1^–/–^* mice via i.v. injection in RPMI (Invitrogen). Mice were infected with *L*. *donovani* 24 hours later, and assessment of parasitic burden was performed at 14 days p.i. In some experiments, *L*. *donovani*–infected C57BL/6J mice were treated with 0.5 mg anti-LAG3 mAb (clone C9B7W; Bio X Cell), anti-TIM3 mAb (clone RMT3-23; Bio X Cell) or control rat IgG (MilliporeSigma) i.p. every 3 days from days 14 to 28 p.i.

### Cell sorting.

MACS purified CD4^+^ cells were restimulated with Phorbol 12-myristate 13-acetate (50 ng/mL, MilliporeSigma) and ionomycin calcium salt (1 μg/mL, MilliporeSigma) in complete RPMI (cRPMI; human: 10% [v/v] fetal bovine serum [Thermo Fisher Scientific], gentamycin [20 μg/mL, MilliporeSigma], RPMI1640 [Invitrogen]; mouse: 10% [v/v] FBS, 1% [v/v] penicillin/streptomycin [Invitrogen], RPMI) at 37°C for 3–5 hours. IL-10 and IFN-γ cytokines were then captured on the surface of the cells using the Miltenyi Biotec IL-10 (PE) and IFN-γ (APC or FITC) Secretion Assays, according to the manufacturer’s guidelines. Cells were then stained with antibodies for 20 minutes, at room temperature, protected from light. IL-10^–^IFN-γ^–^, IL-10^–^IFN-γ^+^, IL-10^+^IFN-γ^+^, and IL-10^+^IFN-γ^–^ cells were sorted from CD8^–^ mouse cells with a BD FACSARIA III (BD Biosciences). Cells were stored at –80°C in 1% (v/v) 2-mercaptoethanol (MilliporeSigma) in RLT buffer (Qiagen).

### RNA isolation.

RNA was isolated from cells using RNeasy Mini Kit and Qiashredders as per manufacturer’s instructions (Qiagen). The Nanodrop ND-1000 UV-Vis Spectrophotometer (Thermo Fisher Scientific) was used to determine RNA concentration and quality.

### RNA-Seq.

RNA isolated from naive, Tr1 and Th1 sorted cells was used to synthesize cDNA using the High-Capacity cDNA Reverse Transcription kit (Applied Biosciences) according to manufacturer guidelines. Libraries were prepared from cDNA. Single-end mRNA-Seq (50 bp reads) was performed on the Illumina HiSeq by the Australian Genome Research Facility (AGRF, VIC, Australia). RNA-Seq data were then processed using the Galaxy platform (https://usegalaxy.org.au/) (71). *FastQC* was used for quality control of data (https://www.bioinformatics.babraham.ac.uk/projects/fastqc/), and reads were mapped to the mouse (GRCm38/mm10) genome using the *STAR* aligner (72). Transcripts were then assembled, and reads per kilobase of transcript per million mapped reads (RPKM) were estimated using *Cufflinks* (73). *Cuffmerge* was used to merge transcript assemblies, and *HTseq* was used to transform mapped reads into counts based on the GENCODE vM9 annotation (74–76). Finally, *EdgeR* was used to analyze these counts for differential gene expression (77).

### Pathway analysis.

Pathway analysis was performed using Ingenuity Pathway Analysis software (Qiagen; content version 81348237; release date: 2022-09-16).

### Comparing Tr1 cell transcriptional signatures from experimental VL and malaria.

DEGs from the Tr1 versus Th1 comparison were compared with 2031 DEGs in Tr1 versus Th1 cells from experimental malaria infection (20). Genes that were differentially expressed in the 2 data sets but in opposing directions based on logFC were excluded. The analysis was performed using the VennDetail package (v1.10.0) (78), and the results were illustrated as a Venn diagram produced using the VennDiagram package (v1.7.3) run on R (v4.1.3) (79).

### Isolation of mouse mononuclear cells and CD4^+^ T cell polarization for ChIP.

Spleen mononuclear cells were isolated from C57BL/6*-Foxp3^tm1flv^*/J (Foxp3-RFP homozygous) × C57BL/6-*Il10^tm1Flv^*/J (IL-10–GFP heterozygous) mice and prepared as previously described (19). Whole spleens were then cultured with anti-CD28 (1 μg/mL, clone 37.51, BioLegend) and plate-bound anti-CD3 (wells coated with 1 μg/mL for 4 hours at 37°C, 5% CO_2_, clone 145-2C11, BioLegend), supplemented with Tr1 cell polarizing cytokines (10 ng/mL IL-2, 10 ng/mL IL-12, 10 ng/mL anti-IL-4 [all from eBioSciences], and 50 ng/mL IL-27 [Thermo Fisher Scientific]) in a 96-well plate (80). After 3 days, cells were harvested and stained with anti-CD4 conjugated to BV421 (BioLegend, clone GK1.5) and anti-CD90.2 conjugated to PerCPcy5.5 (BioLegend, clone Thy1.2); then FoxP3^–^IL-10^+^CD4^+^ T cells were isolated using a BD FACSARIA III (BD Biosciences), prior to preparing cells for ChIP.

### ChIP.

ChIP assays were conducted as previously described (81), with a sheared fragment size of 300 bp to 1 kb, using anti-Pbx1 (clone 4342, Cell Signaling Technology), anti–pol II (clone ab817, Abcam), and rabbit IgG (clone ab37415, Abcam). In total, 1 μL (from 30 μL) of immunoprecipitated DNA was used for quantitative PCR (qPCR). Primers A–F (Figure 5) were designed to amplify –1 to –4.593 kb upstream of the IL-10 transcription start site or –1 to –4,813 kb upstream of the IFN-γ transcription start site (Supplemental Table 4), including regions where 3 PBX1 consensus binding sites exist. Abundance of PBX1 or RNA Pol II binding is illustrated as a percent of immunoprecipitated target sequences relative to input chromatin.

### L. donovani whole parasite splenocyte stimulation.

Mouse splenocytes (1 × 10^5^) were cultured with 2 × 10^6^ fixed *L*. *donovani* amastigotes and anti–mouse LAG3 (Bio X Cell, clone C9B7W) or anti–mouse TIM3 (Bio X Cell, clone RMT3-23) mAbs or rat IgG1 (clone MAC49, made in house) all at 20 μg/mL in cRPMI for 72 hours at 37°C, 5% (v/v) CO_2_. Cell culture supernatant was harvested after 24 hours and stored at –20°C.

### Flow cytometry.

In addition to sorting, flow cytometry was also used to assess cells. Cells were prestained with anti–mouse LAG3 conjugated to BV785 (BioLegend, clone C9B7W), CCR2 conjugated to BV421 (BioLegend, clone SA203G11), CCR5 conjugated to PercPCy5.5 (BioLegend, clone HM-CCR5), TIM3 conjugated to PE (BioLegend, clone B8.2C12), and TIGIT conjugated to APC (BioLegend, clone 1G9) in 50 μL cRPMI for 30 minutes at 37°C, 5% CO_2_, after initial staining, 150 μL of cRPMI supplemented with Monensin (eBioscience, 4 μM final concentration), Phorbol 12-myristate 13-acetate (MilliporeSigma, 50 μg/mL final concentration), and Ionomycin calcium salt (MilliporeSigma, 1 μg/mL final concentration) were added and cells were incubated for 3 hours at 37°C, 5% CO_2_. After stimulation, cells were washed with PBS and then stained with anti–mouse CD45 conjugated to FITC (BioLegend, clone 30-F11), TCRβ conjugated to BUV737 (BD Biosciences, clone H57-597), CD4 conjugated to BUV395 (BD Biosciences, clone GK1.5), CD8 Alexa Fluor 800 (BioLegend, clone 53-6.7), PD-1 conjugated to APC-FIRE750 (BioLegend, clone 29F.1A12), and CD49b conjugated to PECy7 (BioLegend, clone HMα2) in 50 μL for 20 minutes at 37°C, 5% CO_2_. Cells were then washed with PBS and stained with Zombie Aqua fixable viability stain (BioLegend, 0.05 μL/sample) in 50 μL for 10 minutes at room temperature. Cells were then washed with PBS, and 100 μL of Cell Fix (eBioscience Foxp3 Transcription factor staining buffer set) was added for 20 minutes at 4°C; cells were washed with Perm/Wash Fuffer (eBioSciences) and stained with anti–mouse CTLA-4 conjugated to BV605 (BioLegend, clone UC10-4B9), IFN-γ conjugated to BV650 (BD Biosciences, clone XMG1.2), IL-10 conjugated to PE-Dazzle594 (BioLegend, clone JES5-16E3), T-bet conjugated to eFluor660 (eBioscience, clone eBio4B10), and Foxp3 conjugated to eFluor450 (eBioscience, clone FJK-16s) for 45 minutes at 37°C, 5% CO_2_. All surface stains were made up in PBS, and all intracellular stains were made up in Perm/Wash Buffer (eBioscience). Samples were acquired in a Cytek Aurora 5 within 24 hours after staining.

### Cytokine analysis.

CD4^+^ T cells were isolated by MACS from the spleens of *Pbx1*^ΔT^ and *Pbx1^fl/fl^* mice, as described above, and cultured for 72 hours with no stimulation or under Th1 or Tr1 cell polarizing conditions, as previously described (82). Cell culture supernatants were then collected for measurement of IFN-γ and IL-10 levels. Cytokine levels were assessed using the BD Cytometric Bead Array (CBA) Mouse Inflammation Kit or Mouse Th1/Th2/Th17 Cytokine Kit (BD Biosciences) as per manufacturer instructions. Serum samples from mouse blood were used undiluted, while cell culture supernatants were diluted 1:5 in 1× PBS for the detection of most cytokines. Supernatants were diluted 1:50 in 1× PBS for the detection of IFN-γ. CBA data were analyzed using the FCAP Array Software v3.0 (BD Biosciences). Human IFN-γ in whole-blood assays was measured by ELISA, as previously described (54).

### qPCR.

RNA was extracted from human PBMCs or CD4^+^ T cells enriched by MACS (Miltenyi Biotec) according to manufacturer instructions. RNA was reverse transcribed to cDNA as previously described (39). qPCR for *LAG3* and *HAVCR2* (encoding TIM3) was performed on an ABI Prism 7500 real-time PCR system (Applied Biosystems) using the TaqMan Gene Expression Assay (Assay ID: Hs01120688_g1; Applied Biosystems). Relative quantification was performed using the ΔΔCT method (83) relative to 18S rRNA (Assay ID: Hs99999901_s1; Applied Biosystems).

### Statistics.

Statistical analysis was performed using GraphPad Prism 6 (GraphPad Software). Analysis of human cellular assays was performed using Wilcoxon matched-pairs signed rank test or nonparametric Mann-Whitney *U* tests, as appropriate. Analysis of mouse data used Mann-Whitney *U* tests for comparisons between 2 groups and 1-way ANOVA to assess more than 2 groups within an experiment. *P* < 0.05 was considered significant.

### Study approval.

The study using human samples was approved by the Ethics Committee of the Institute of Medical Sciences, Banaras Hindu University, and all patients provided written informed consent. Experimental animal use was in accordance with the “Australian Code of Practice for the Care and Use of Animals for Scientific Purposes” (Australian National Health and Medical Research Council [NHMRC]) and approved by the QIMR Berghofer Medical research Institute Animal Ethics Committee (Herston QLD, Australia; approval no. P2304).

### Data availability.

All data are available in the main text and supplementary materials. The mouse RNA-Seq data are available in the Gene Expression Omnibus (GEO) database (https://www.ncbi.nlm.nih.gov/geo/) under accession no. GSE139933. Flow cytometry analysis was performed using OMIQ Data science platform (https://www.omiq.ai), including tSNE dimension reduction, Flow Self organizing maps (FlowSOM) clustering, edgeR statistical analysis, and heatmap generation. Values for all data points in graphs are reported in the Supporting Data Values file.

## Author contributions

Conceptualization was contributed by CLE, SSN, SN, SS, RK, and CRE conceptualized the study. CLE, JAE, FDLR, SSN, DC, MMDO, TCMF, SBC, SSS, AK, YW, JN, PM, JSL, SN, and RK developed the methodology and performed experiments and human studies. SN, SS, and CRE acquired the funding for the research. SSS, RK, and CRE administered the project. FDLR, MMDO, SN, SS, RK, and CRE supervised the research. CLE and CRE wrote the original draft manuscript. CLE, SSN, JAE, RK, and CRE reviewed and edited the paper.

## Supplementary Material

Supplemental data

Supplemental table 1

Supplemental table 2

Supporting data values

## Version 1 11/02/2023

In-Press Preview

## Version 2 12/22/2023

Electronic publication
