# Supplementary material for: A molecular signature for IL-10–producing Th1 cells in protozoan parasitic diseases
Source: JCI Insight. 2023 Dec 22;8(24):e169362. doi: 10.1172/jci.insight.169362 (PMC10807716; doi:10.1172/jci.insight.169362)
Supplement: Supplemental data [file jciinsight-8-169362-s136.pdf]

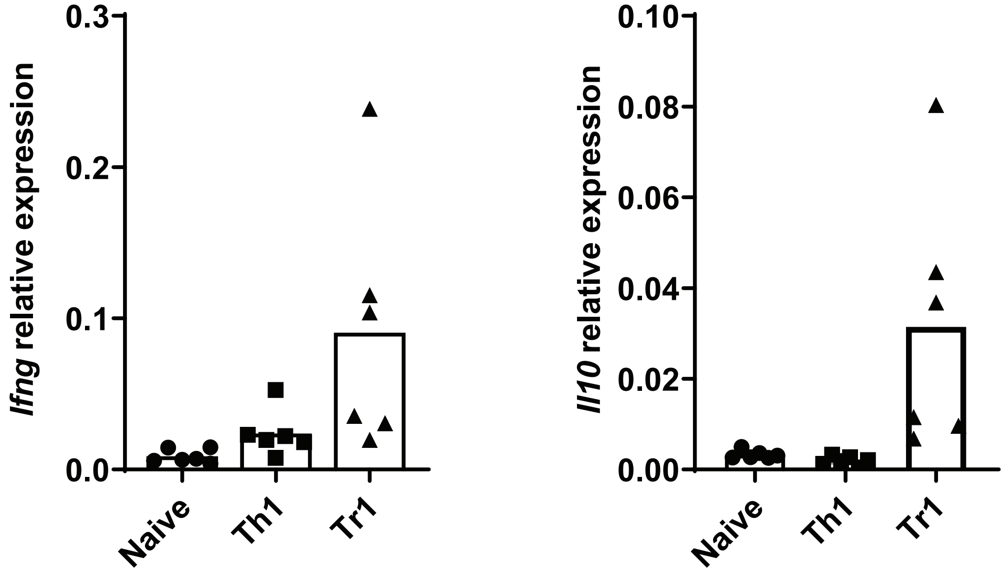

Figure S1. Related to Figure 1. *Ifng* and *Il10* mRNA expression in sorted splenic CD4$^+$ T cell populations from mice infected with *Leishmania donovani* for 14 days. Cytokine-negative (Naïve), Th1 and Tr1 cells were isolated as outlined in Figure 1, n = 6 paired samples.

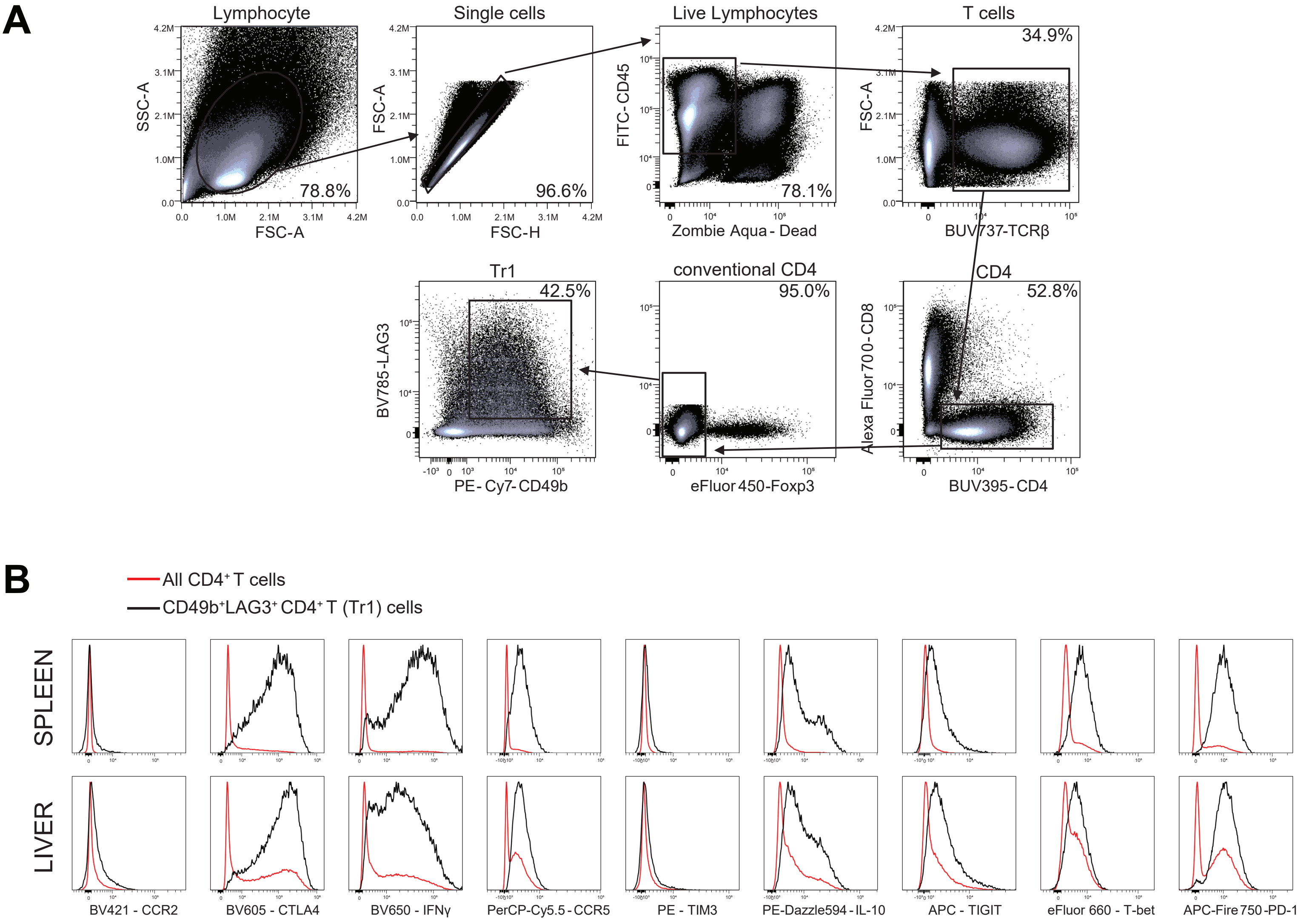

Figure S2. Gating strategy for identification of Tr1 cells in the liver during experimental visceral leishmaniasis caused by *Leishmania donovani*. Tr1 cells defined by high levels of CD49b and LAG3 expression were assessed by FACS at day 14 post-infection (A). The expression of CCR2, CTLA4, IFNγ, CCR5, TIM3, IL-10, TIGIT, T-bet and PD-1 on all $CD4^+$ T cells (red line) and Tr1 cells (black line) was measured in liver and spleen on day 14 post-infection, with data shown as concatinated histograms (n =5 paired samples) (B).

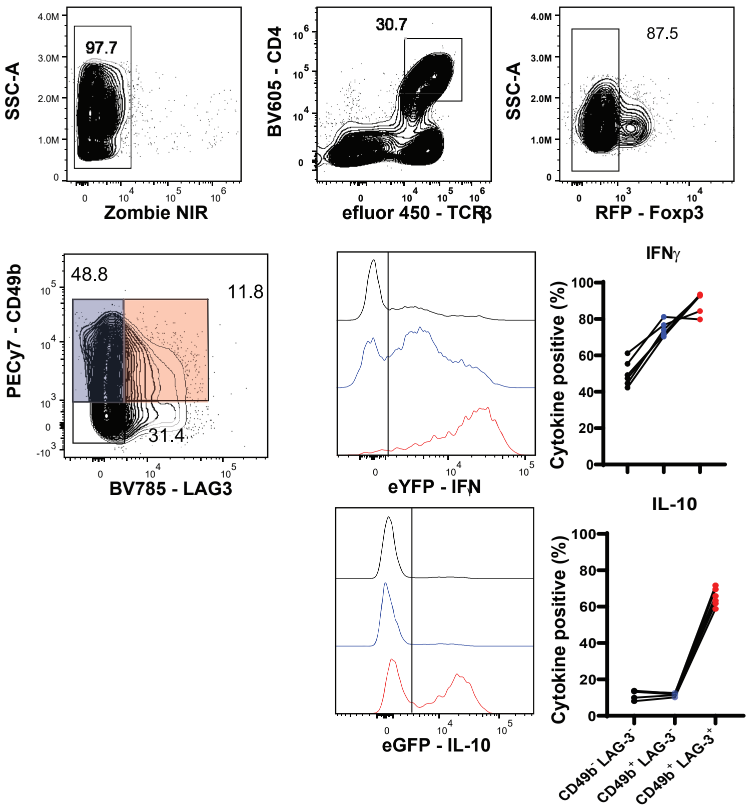

Figure S3. IFNγ^{+} IL-10^{+} CD4^{+} T (Tr1) cells are CD49b^{+} LAG3^{+}. *Il10gfp* x *Ifngyfp* x *Foxp3rfp* mice were infected with 2x10^{7} *L. donovani* amastigotes intravenously and 14 days post-infection (p.i.), conventional (*Foxp*3^{-}) CD4^{+} T cells were assessed by flow cytometry for CD49b and LAG3 expression and co-expression of *Il10gfp* and *Ifngyfp.* The frequency of IFNγ^{+} and IL-10^{+} amongst CD49b and LAG3 expressing CD4^{+} T cells is shown.

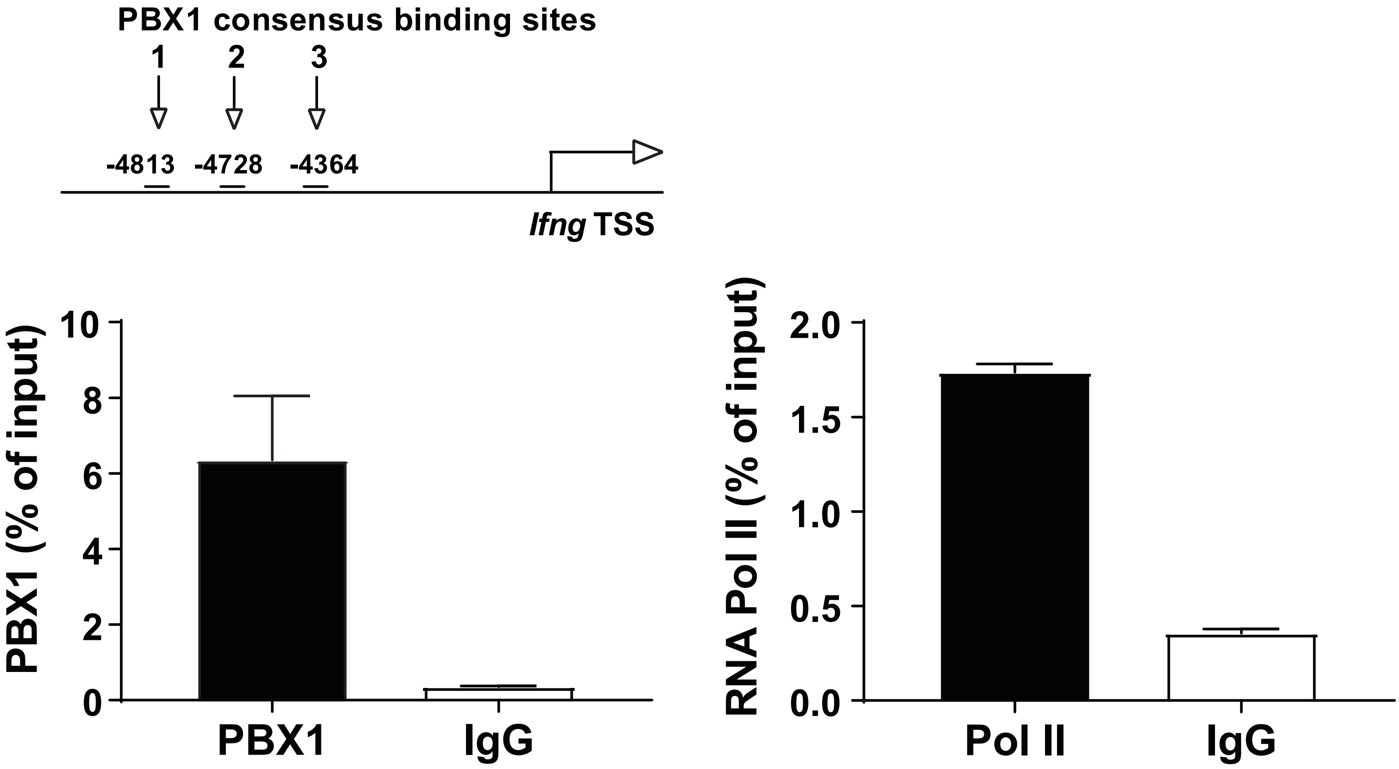

Figure S4. PBX1 binds to the *Ifng* promoter region. Tr1 cells were sorted from mouse splenocytes which had been cultured in Tr1 cell polarizing conditions. Tr1 cells were assessed via chromium immunoprecipitation (ChIP) for PBX1 and RNA polymerase II (RNA Pol II) binding upstream of the *Ifng* transcription start site (TSS) in the promoter region. PBX1 consensus binding sites in the *Ifng* promoter are indicated. Recruitment of PBX1 and RNA Pol II to the PBX1 consensus binding sites are shown (A). Mean ± SEM.

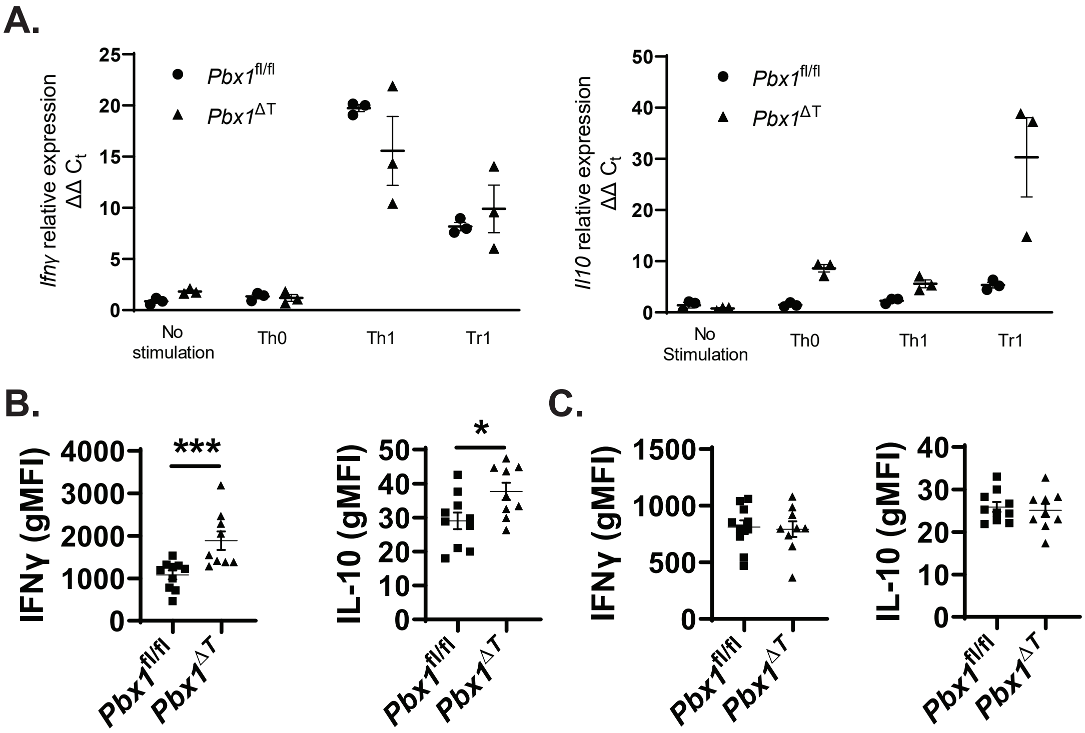

Figure S5. ***Ifn*γ and *Il10* expression by CD4+ T cells in the absence of *Pbx1*.** Splenic CD4$^+$ T cells were isolated from *Pbx1$^{fl/fl}$* and *Pbx1$^{\Delta T}$* mice and cultured for 72 hours under the polarizing conditions described. RNA was prepared and *Ifn*γ and *Il10* expression measued by qPCR, as indicated. n = 3 individual mice from each genotype (A). *Pbx1$^{\Delta T}$* (n = 10) and litter mate control mice (*Pbx1$^{fl/fl}$*; n = 9) were infected with *Leishmania donovani* for 14 days prior to measuring CD4$^+$ T cell IFNγ and IL-10 geometric mean fluorescent intensity (gMFI) in the liver (B) and spleen (C). The gating used for analysis was as shown in Supplementary Figure 2. Mean ± SEM, *p<0.05, and ***p<0.001; significance assessed by Mann-Whitney test.

Table S3. Clinical data for visceral leishmaniasis patient and endemic control participants.

Variables	VL	EC
**N**	30	12
**Age (years)**	27.0±15.22 (22.5)[a]	34.3±6.99 (35)
**Sex (M/F)**	16/14	5/7
**Duration of illness (days)**	37.03±28.41 (30)	N/A
**WBC (D-0)**	4550±2764.02 (3600)	N/D
**WBC (D-Dis)**	7826.66±2901.95 (7150)	N/D
**Platelets (×10^3/mm3,D-0)**	104.466±60.010 (94)	N/D
**Platelets (×10^3/mm3,D-Dis)**	207.566±80.38 (189)	N/D
**Splenic enlargement (cm, D-0)**	2.83±1.68 (3)	N/A
**Splenic enlargement (cm, D-Dis)**	0.23±0.62 (0)	N/A

Abbreviations: N/A, not applicable; ND, not done.

[a]Mean values ± SD of aggregated data are shown, and median values are in parentheses.

Table S5. Primers used for Chromatin immunoprecipitation.

Target region	Forward primer (5' to 3')	Reverse primer (3' to 5')
hprt	CCTAAATCTTGAGGAATCACATCA	TTCTTTCTGAAGAAAATGGTACTGG
il10 A	GAAGAAAATCAGCCCTCTCG	GGATAAATGGGCTATTCCAGA
il10 B	CAGTCAGGAGAGAGGGCAGT	TTTTGGGATGTCCGTTTCC
il10 C	AAGAGGTGCTGCTTCTCCTG	TGGCACTGGACAGTTCTATGA
il10 D	GGGCTTGATAACGTGTGAGTG	AAGAAGTTCCCCCTGTAGCTG
il10 E	AACACTCTTCAGGGGTGAGC	AGTCTGGGAGCTCTGAGTGG
il10 F	GGTTATCTGGGGTAGTCATGGA	TGTGGAATTCCCCTTTGTGT
Ifng	ATTTGGCCCAATTGTATGGA	CACAGCATGTCATGACCACA
